# Status of the neonatal follow-up system in China: survey and analysis

**DOI:** 10.1007/s12519-023-00742-6

**Published:** 2023-07-15

**Authors:** Qi Zhou, Yun Cao, Lan Zhang, Nurya Erejep, Wen-Long Xiu, Jing-Yun Shi, Rui Cheng, Wen-Hao Zhou, Shoo K. Lee

**Affiliations:** 1 Department of Neonatology, Children’s Hospital of Fudan University, 399 Wanyuan Rd, Minhang District, Shanghai 201102, China; 2 Department of Neonatology, Children’s Hospital of Xinjiang, Urumqi, China; 3 Department of Neonatology, Fujian Maternity and Child Health Hospital College of Clinical Medicine for Obstetrics & Gynecology and Pediatrics, Fujian Medical University, Fuzhou, China; 4 Department of Neonatology, Gansu Provincial Maternal and Child Care Hospital, Lanzhou, China; 5 Department of Neonatology, Nanjing Children’s Hospital, Nanjing, China; 6 Department of Pediatrics, Mount Sinai Hospital, Toronto, ON Canada

**Keywords:** Follow-up, Neonate, Survey

## Abstract

**Background:**

There is little information about neonatal follow-up programs (NFUPs) in China. This study aimed to conduct a survey of hospitals participating in the Chinese Neonatal Network (CHNN) to determine the status of NFUPs, including resources available, criteria for enrollment, neurodevelopmental assessments, and duration of follow-up.

**Methods:**

We conducted a descriptive study using an online survey of all 72 hospitals participating in CHNN in 2020. The survey included 15 questions that were developed based on the current literature and investigators’ knowledge about follow-up practices in China.

**Results:**

Sixty-four (89%) of the 72 hospitals responded to the survey, with an even distribution of children’s (31%), maternity (33%) and general (36%) hospitals. All but one (98%) hospital had NFUPs, with 44 (70%) being established after 2010. Eligibility criteria for follow-up were variable, but common criteria included very preterm infants < 32 weeks or < 2000 g birth weight (100%), small for gestational age (97%), hypoxic ischemic encephalopathy (98%) and postsurgery (90%). The average follow-up rate was 70% (range: 7.5%–100%). Only 12% of hospitals followed up with patients for more than 24 months. There was significant variation in neurodevelopmental assessments, follow-up schedule, composition of staff, and clinic facilities and resources. None of the staff had received formal training, and only four hospitals had sent staff to foreign hospitals as observers.

**Conclusions:**

There is significant variation in eligibility criteria, duration of follow-up, types of assessments, staffing, training and facilities available. Coordination and standardization are urgently needed.

**Supplementary Information:**

The online version contains supplementary material available at 10.1007/s12519-023-00742-6.

## Introduction

Neonatal intensive care has developed rapidly in China during the past few decades, and neonatal mortality decreased from 30/1000 live births in 1989 to 4/1000 live births in 2020 [1]. Survival of extremely preterm infants has also increased, and Zhu et al. reported that survival of infants born at < 28 weeks gestational age (GA) increased from 56.4% in 2010 to 68% in 2019 [2], with a concomitant increase in the incidence of major morbidities from 52% in 2010 to 72.4% in 2019. At the same time, preterm birth rates have risen steadily. Zhang et al. reported that preterm birth rates rose by 1.1% each year between 1990 and 2016 [3]. This has led to an increasing recognition of the importance of standardized neonatal outcome surveillance, including neonatal follow-up and neurodevelopmental assessment, and many neonatal intensive care units (NICUs) in China have established neonatal follow-up programs [4, 5]. Neonatal follow-up programs are important because they permit comprehensive evaluation of the outcomes of neonatal-perinatal care, initiation of early intervention programs to improve infant development outcomes, implementation of quality improvement measures, and improvement of health care delivery.

Some countries have established national guidelines for neonatal follow-up using standardized tools, but this has not yet happened in China [6–8]. To date, the development of neonatal follow-up programs in China has not been adequately described. In 2013, Sun et al. reported that many provincial and sub-provincial maternity and children's hospitals have established neonatal follow-up programs and rehabilitation clinics, especially for those recovering from initial brain injury, but they did not provide specifics about the number of clinics, assessments made or interventions provided [5]. Hu et al. published a protocol used in a hospital in Guangzhou [9], but since neonatal follow-up programs in China developed independently at different hospitals with little coordination or standardization, it is unclear how prevalent neonatal follow-up programs are, which infants are assessed and when, what assessment tools are used or the training received, and what resources are available.

The Chinese Neonatal Network (CHNN) was established in 2018 as a national collaboration of NICUs, neonatologists and nurses to improve the training, research, clinical care and outcomes of newborn infants in China. It currently comprises 92 tertiary level NICUs in 31 provinces across China, which participate in standardized outcome surveillance and benchmarking, collaborative research and quality improvement [4, 10, 11]. The 92 participating NICUs include all government-designated neonatal centers of excellence in China, including four national children’s medical centers, five regional children’s medical centers, and 30 provincial perinatal or children’s medical centers. Other hospitals comprise major referral centers in large cities across China. CHNN establishes and maintains a standardized database of short-term in-hospital outcomes for very preterm infants with GA < 32 weeks or with birth weight < 1500 g, as well as clinical practices in participating NICUs, and conducts collaborative research aimed at improving quality of care. An annual data quality audit using reabstraction of randomly selected charts reported high quality of data collected [12]. Anecdotally, many of the hospitals participating in CHNN also have neonatal follow-up programs for high-risk infants discharged from their NICUs, but there is little information on them.

The objective of this study was to conduct a survey to determine the status of neonatal follow-up programs in CHNN hospitals, including the prevalence of neonatal follow-up programs, resources available, criteria for infant enrollment, schedule and type of neurodevelopmental assessments, and duration of follow-up.

## Methods

### Descriptive study and participants

We conducted a descriptive study using an online survey of all 72 hospitals that were participating in CHNN in 2020. The survey included 15 questions that were developed based on current literature and investigators’ knowledge about follow-up practices in China [13]. Themes included presence or absence of a neonatal follow-up program, resources available, eligibility criteria, staffing models, services provided, timing and frequency of follow-up visits, the range of assessments conducted at each visit, and the follow-up rate (Supplementary Table 1). The survey was focused mainly on follow-up during the first 5 years post discharge, as few Chinese NICUs follow-up infants beyond that duration. The questions were piloted at a single center, and modifications were made following their feedback.

### Data analyses and ethics

Data were analyzed descriptively and tabulated. The study was approved by the institutional ethics board of Children’s Hospital of Fudan University (CHFU 2018–296) and all participating sites.

## Results

Sixty-four (89%) (Supplementary Table 2) of the 72 hospitals responded to the survey (Table 1). There was an even distribution of children’s (31%), maternity (33%) and general (36%) hospitals. All regions of the country were represented, including 27 provinces and 40 cities. Although 47% of the hospitals were from East China, it should be noted that this is where the majority of the population is. All but one (98%) hospital had a neonatal follow-up program, with 58 (92%) being established after 2000 and 44 (70%) after 2010. Eligibility criteria for follow-up were variable, but very preterm infants < 32 weeks or < 2000 g birth weight were seen by all hospitals. Other common eligibility criteria were small for GA (97%), hypoxic ischemic encephalopathy (98%) and postsurgery (90%). Patients with hyperbilirubinemia were only followed up by 38% of hospitals, and intracranial injuries (intraventricular hemorrhage, intracranial hemorrhage, intracranial infections) were followed up by 32% of hospitals. The average follow-up rate was 70% (range: 7.5%–100%).

**Table 1:** Geographical and organizational characteristics of the neonatal follow-up clinics

Characteristics	*n/N*	%
Region		
East China	30/64	46.9
North China	6/64	9.4
South China	7/64	10.9
Central China	6/64	9.4
Northeast China	3/64	4.7
Southwest China	6/64	9.4
Northwest China	6/64	9.4
Hospital type		
General hospital	23/64	35.9
Children's hospital	20/64	31.2
Maternal infant care hospital	21/64	32.8
Neonatal follow-up clinic available		
Yes	63/64	98.4
No	1/64	1.6
Year of establishment of neonatal follow-up clinic		
1980s	2/63	3.2
1990s	3/63	4.8
2000s	14/63	22.2
2010s	43/63	68.2
2020s	1/63	1.6
Criteria for neonatal follow-up		
Very premature (≤ 32 wk and/or ≤ 2000 g)	63/63	100.0
Small for gestational age	61/63	96.8
Hypoxic ischemic encephalopathy infant	62/63	98.4
Post-surgery	57/63	90.5
Hyperbilirubinemia	24/63	38.1
Intracranial infection	20/63	31.7
Intraventricular/intracranial hemorrhage	19/63	30.2
Inherited metabolic disease	2/63	3.2
Congenital heart disease	2/63	3.2
Extra-corporeal membrane oxygenation	2/63	3.2
Estimated number of follow-up patients annually		
≥ 1000	28/63	44.4
500–999	14/63	22.2
< 500	21/63	33.3
Estimated follow-up rate		
≥ 70%	41/63	65.1

Patients were followed up for less than 18 months by 30 (48%) hospitals, for up to 24 months by 25 (40%) hospitals, and for more than 24 months by only 8 (12%) hospitals (Table 2). The most commonly used assessment tools were the Neonatal Behavior Neurological Assessment (NBNA) (81%, *n* = 51), General Movement Assessment (GMA) (73%, *n* = 46), Bayley Scales of Infant & Toddler Development (BAYLEY) (68%, *n* = 43) and Ages & Stages Questionnaire Chinese Version (ASQ-C) (61%, *n* = 39). Physical, neurological, nutritional and physiotherapy examinations were performed by all hospitals. Visual, hearing, occupational and behavioral assessments were performed by 90% (*n* = 57), 96% (*n* = 61), 92% (*n* = 58) and 85% (*n* = 54), respectively.

**Table 2:** Assessments at neonatal follow-up clinics based on the 63 hospitals

Characteristics	*n/N*	%
Follow-up endpoint		
CA < 18 mon	30/63	47.6
CA 18–24 mon	25/63	39.7
CA > 24 mon	8/63	12.7
Follow-up assessment tools		
Neonatal behavior neurological assessment	51/63	81.0
General movement assessment	46/63	73.0
Bayley scales of infant & toddler development	43/63	68.2
Ages & stages questionnaire	39/63	61.9
Amiel-Tison neurologic evaluation	35/63	55.6
Hammersmith infant neurological examination	17/63	27.0
Alberta infant motor scale for gross motor	31/63	49.2
Posture and fine motor assessment of infants	24/63	38.1
Communication symbolic behavior scales infant/toddler checklist	29/63	46.0
Peabody developmental motor scale	29/63	46.0
Griffith mental development scales	10/63	15.9
Developmental scale for 0–6 y	4/63	6.3
Denver development screening test	3/63	4.8
Test of infant motor performance	3/63	4.8
Infant neurological international battery	1/63	1.6
Modified check list for autism in toddlers	38/55	69.1
Child behavior checklist	30/55	54.5
Parenting stress index short form	8/55	14.5
Follow-up assessment		
Physical examination	63/63	100.0
Neurological examination	63/63	100.0
Growth	63/63	100.0
Visual	57/63	90.5
Hearing	61/63	96.8
Psychology assessment	39/63	61.9
Physiotherapy assessment and intervention	63/63	100.0
Occupational therapy assessment and intervention	58/63	92.1
Behavior therapy and intervention	54/63	85.7

*CA* corrected age

Follow-up clinic staffing included doctors 100% (*n* = 63), nurses 82% (*n* = 52), physiotherapists 52% (*n* = 33), occupational therapists 73% (*n* = 46), and psychologists 27% (*n* = 17) (Table 3). None of the staff in participating hospitals received formal training in neonatal follow-up. Sixty-eight (*n* = 43) percent of hospitals reported that their staff received some form of informal training or observation at other sites, 63% (*n* = 40) reported that training was less than 1 year in duration, and four hospitals sent staff to attend neonatal follow-up programs at foreign hospitals as observers. The follow-up clinic was a combined clinic in 93% (*n* = 59), with developmental pediatric involvement in 80% (*n* = 51), a rehabilitation department in 79% (*n* = 50) and a psychology department in 28% (*n* = 18). Follow-up clinic facilities included dedicated examination rooms in 98% (*n* = 62), rooms with one-way mirrors in 15% (*n* = 10), and video capability in 36% (*n* = 23). Virtual assessments were performed by 40% (*n* = 25).

**Table 3:** Neonatal follow-up clinic resources based on the 63 hospitals

Characteristics	*n/N*	%
Follow-up clinic staffing		
Doctor	63/63	100.0
Nurse	52/63	82.5
Physiotherapist	33/63	52.4
Occupational therapist	46/63	73.0
Psychologist	17/63	27.0
Combined follow-up clinic (with another department)	59/63	93.6
Developmental pediatrics	51/63	81.0
Rehabilitation	50/63	79.4
Psychology	18/63	28.6
Follow-up clinic facilities		
Examination room	62/63	98.4
Room with 1-way mirror	10/63	15.9
Video capability	23/63	36.5
Virtual assessment		
Yes	25/63	39.7
No	38/63	60.3

Commonly used follow-up time points for follow-up were 40 weeks, 3 months, 6 months, 12 months, 18–24 months and 36 months corrected age, with 12 months and 18–24 months CA being the most commonly used time points (Table 4). A variety of different assessment tools were used at the different time points. All 64 hospitals indicated that they were interested in joining a Chinese Neonatal Follow-up Network.

**Table 4:** Assessment tools used at various follow-up time points based on the 55 hospitals

Time point	Physical	NBNA	ASQ	GMS	ANTA	HINE	AIMS	PFMA	CSBS	GMDS	BAYLEY	CBCL	PSI	MCHAT
40 wk	55 (100.0)	48 (87.3)	NA			NA					NA	NA		NA
3 mon	55 (100.0)	NA	33 (60.0)	35 (63.6)	15 (27.3)	11 (20.0)	26 (47.3)	15 (27.3)			NA	NA		NA
6 mon	55 (100.0)	NA	36 (65.4)	NA	19 (34.5)	12 (21.8)	27 (49.1)	20 (36.4)	10 (18.2)		NA	NA	6 (10.9)	NA
12 mon	55 (100.0)	NA	38 (69.1)	NA	15 (27.3)	11 (20.0)	22 (40.0)	25 (45.4)	10 (18.2)		NA	NA	6 (10.9)	NA
18–24 mon	55 (100.0)	NA	34 (61.8)	NA						18 (32.7)	34 (61.8)	24 (43.6)	8 (14.5)	24 (43.6)
36 mon	53 (96.4)	NA	NA	NA		NA	NA		NA	16 (29.1)	33 (60.0)	26 (47.3)	9 (16.4)	NA

Data are presented as *n* (%). *NBNA* neonatal behavioral neurological assessment, *ASQ* ages & stages questionnaire, *GMS* general movement scale, *ATNA* Amil-Tison neurological assessment, *HINE* Hammersmith infant neurological examination, *AIMS* Alberta infant motor scale, *PFMA* pediatric fine motor assessment, *CSBS* communication and symbolic behavior scales developmental profile, *GMDS* Griffith mental developmental scales, *BAYLEY* Bayley scales of infant development, *CBCL* child behavior check list, *PSI* parenting stress index short form, *MCHAT* modified check list for autism in toddlers revised with follow-up (Chinese version), *NA* not applicable

## Discussion

This is the first comprehensive study of the status of neonatal follow-up programs in China. Although the study only included 64 of the 72 hospitals participating in CHNN in 2020, they represent major hospitals with tertiary level NICUs in China. Our study shows that most of the follow-up programs in China were established only recently, with 92% being established after 2000 and 70% being established after 2010. This demonstrates an awareness of the importance of neonatal follow-up programs in China, and NICUs are endeavoring to provide this service. Indeed, only one hospital of the 64 hospitals that responded to the survey did not have a neonatal follow-up program at the time of the survey.

The average neonatal follow-up rate of 70% was respectable, although most neonatal follow-up programs strive to achieve 90%. In China, neonatal follow-up is challenging because the lack of a perinatal regionalization system and the competitive financial environment in which Chinese hospitals operate mean that not all patients return to referral hospitals for neonatal follow-up. The market-oriented health care system in China also means that patients may misinterpret neonatal follow-up as a means for the hospital to maximize revenue through a service that is of little benefit to the patient. Communication with families is therefore essential for promoting trust and encouraging participation in neonatal follow-up.

There was significant variation among the follow-up programs. Eligibility criteria varied, but there was a common focus on preterm infants born at < 32 weeks GA, small for gestational age, hypoxic ischemic encephalopathy and post-surgical patients. In the majority of hospitals, the duration of follow-up was short, with 88% of hospitals only following up with patients for 24 months. Twenty-four months is often considered to be the earliest age at which neurodevelopmental testing is sufficiently stable for prognostication. However, more recently, Taylor et al. reported that among children classified as having moderate to severe neurodevelopmental impairment (NDI) at 2 years, 63% had none to mild NDI at 10 years; among children classified as having profound NDI at 2 years, 36% had no to mild NDI at 10 years [14]. Consequently, it is important to follow-up infants for longer than 2 years, as neurodevelopmental outcomes can still improve beyond that time.

There was significant variation in the types of follow-up assessment instruments used. Among the more commonly used instruments, the NBNA was used by 81% (*n* = 51), GMA by 73% (*n* = 46), BAYLEY by 68% (*n* = 43) and ASQ-C by 61% (*n* = 39) [15–18]. The NBNA is a domestic instrument developed by Bao et al. in 1991 based on the method of Brazelton and Amiel-Tison [19], as well as their own experience in China, for behavioral neurological measurement in newborns. This instrument has been validated in China for predicting the prognosis of term asphyxiated infants (sensitivity: 84.6%, specificity: 97.4%) at 12–14 days [20]. The NBNA assesses functional abilities, reflexes and responses, and stability of behavioral status in five clusters: behavior, passive tone, active tone, primary reflexes, and general assessment. GMA has been used to assess spontaneous general movements of infants from preterm to approximately 5 months post term age [20]. GMA (sensitivity: 98%, specificity: 91%) has been proven to be one of the tools with the best predictive validity for the early detection of cerebral palsy, particularly at 3–5 months of age. The GMA has been shown to be reliable and valid for the Chinese population, with high sensitivity, specificity, inter-scorer reliability and test–retest reliability [21]. BAYLEY is a well-known psychometric instrument that provides useful information for the early identification of infants who might have developmental problems [22]. It is the most commonly used test in infant developmental assessments internationally. The BAYLEY scores across five subdomains: cognitive, language, motor, social-emotional and adaptive behavior. The Chinese version has been validated and used in Taiwan Province of China [23], and is currently being validated in mainland of China. The ASQ-C is a screening tool for infant neurodevelopmental evaluation at different corrected ages. The ASQ-C was tested and validated in mainland of China using a representative national sample aged 1–66 months and demonstrated to be a reliable and valid measure [24]. It can effectively screen five key developmental areas: communication, gross motor, fine motor, problem solving, and personal-social. These instruments may therefore form a common base for building a standardized protocol for neonatal follow-up in China.

There was a lack of systematic training for neonatal follow-up. Sixty-eight percent of hospitals reported that their staff only received informal training, mostly through observation at other hospitals for less than 1 year duration, and the remaining hospitals were self-trained. Only four hospitals sent their staff abroad as observers to establish follow-up programs. Training is important to ensure the quality of developmental assessments and should be a priority for the future. Otherwise, prognostication and interventions may be baseless, and comparison and benchmarking of outcomes become meaningless.

Embrace of the multidisciplinary team concept for assessing neurodevelopment in infants appears to be mixed. While physicians (100%), nurses (82%) and occupational therapists (73%) are commonly used in follow-up clinics, physiotherapists (52%), psychologists (26%) and behavioral therapists (0%) are less frequently or not used. Consequently, there is room for encouraging the multidisciplinary concept. There is varied involvement of different hospital departments (neonatology, developmental pediatrics, rehabilitation, psychology) in neonatal follow-up, but they are not always coordinated or work as a single team, which would streamline the process for assessment, intervention and referral. Most hospitals have basic facilities (e.g., dedicated examination rooms) for neonatal follow-up but lack facilities for more in-depth examinations (e.g., video capability, rooms with one-way mirrors).

Many developed countries, such as the UK, Canada and Australia, have adopted national guidelines with common criteria and standardized tools for neonatal follow-up [6–8]. Guidelines should be comprehensive, practical, achievable, affordable, cost-effective and adapted to the country’s needs. The results of this survey are informative and will be used by CHNN to create a national neonatal follow-up guideline suited to the needs of China.

In summary, neonatal follow-up programs are currently available in most tertiary level hospitals with NICUs in China, but there is significant variation in eligibility criteria, duration of follow-up, types of assessment instruments used, staffing, training and facilities available. Standardization of neonatal follow-up protocols and resources would be a major step forward and should be a priority for CHNN.

### Supplementary Information

Below is the link to the electronic supplementary material.

**Supplementary file 1** (PDF 53 KB)

**Supplementary file 2** (MP4 144129 KB)

## Data Availability

All data generated or analyzed during this study are included in this manuscript. Further inquiries can be directed to the corresponding author.
