# Supplementary material for: Status of the neonatal follow-up system in China: survey and analysis
Source: World J Pediatr. 2023 Jul 15;19(11):1104–10. doi: 10.1007/s12519-023-00742-6 (PMC10533627; doi:10.1007/s12519-023-00742-6)
Supplement: Supplementary file 1 — (PDF 53 KB) [file 12519_2023_742_MOESM1_ESM.pdf]

Supplementary Table 1. Chinese neonatal network follow up clinic survey

Name:

Hospital:

City:

Province:

1. Do you have a neonatal follow-up clinic? Yes No

    If No, are you interested in establishing a neonatal follow-up clinic? Yes No

    (then skip to Question 15)

    If Yes, continue below.

2. When was your follow-up clinic started? _______ (year)

3. What are the criteria for babies to come to your follow-up clinic?

    Birth weight: _______ grams

    Gestational age: _______ weeks

    Surgery: Yes No

    Cardiac: Yes No

    Small for Gestational Age: Yes No

    Hypoxic Ischaemic Encephalopathy Yes No

    Others: ____

4. Estimated number of babies meeting criteria in your NICU each year: ________

5. Estimated number of babies seen at your clinic each year: ________

6. At what ages do you see babies in the follow-up clinic?

    First visit: _____ years _____ months

    Second visit: _____ years _____ months

    Third visit: _____ years _____ months

    Fourth visit: _____ years _____ months

    Fifth visit: _____ years _____ months

    Others ____

7. What developmental assessment instruments do you use in the follow-up clinic?

Ages & Stages	Yes	No
General Movement Assessment (GMA/GMS)	Yes	No
Amiel-Tison Neurologic Evaluation	Yes	No
HAMMERSMITH Infant Neurological Examination	Yes	No
Alberta Infant Motor Scale for gross motor (AIMS)	Yes	No
Posture and Fine Motor Assessment of Infants	Yes	No
Communication Symbolic Behavior Scales Infant/Toddler Checklist (CSBS)	Yes	No
Griffith Mental Development Scales (GMDS)	Yes	No
Peabody Developmental Motor Scale (PDMS-2)	Yes	No
Bayley Scales of Infant & Toddler Development	Yes	No

Others: ____

8. What other assessments do you perform?

Physical examination	Yes	No
Neurological examination	Yes	No
Nutrition	Yes	No
Visual	Yes	No
Hearing	Yes	No
Psychologist	Yes	No
Physiotherapy	Yes	No
Occupational therapy	Yes	No
Behavior therapy	Yes	No

Others: ____

9. What staff do you have in your follow-up clinic?

Doctor	Yes	No
Nurse	Yes	No
Physiotherapist	Yes	No
Occupational therapist	Yes	No
Psychologist	Yes	No

Other: ____

10. What formal training did your follow-up doctor receive for neonatal follow-up?

Fellowship training in established neonatal follow-up program <1 yr ≥1 yr

Observation in established neonatal follow-up program <1yr ≥1 yr

Training course in developmental assessment (e.g. Bayley's) Yes No

Other training ____

No formal training [ ] Tick

11. Where was the follow-up training received? What was the training content?

In China _____________________ (Hospital/City) _______________________(content)

Outside China _____________________ (Hospital/Country) ____________________(content)

12. Will a formal neonatal follow-up training program in China be useful? Yes No

13. What facilities do you have in the follow up clinic?

Examination room Yes No

Room with 1-way mirror Yes No

Video capability Yes No

14. Do you perform virtual assessments? Yes No

If so, in what way: Phone call Yes No

Email Yes No

Video Yes No

15. Are you interested in joining a Chinese Neonatal Follow-Up Network and database? Yes No

THANK YOU.

Supplementary Table 2. Hospitals participating in the survey

No.	**Hospitals**
1	Shanghai Children's Medical Center
2	The First Bethune Hospital of Jilin University
3	Dehong People’s Hospital of Yunnan Province
4	Shenzhen Maternity and Child Health Care Hospital
5	First Affiliated Hospital of Xi’an Jiaotong University
6	People’s Hospital of Xinjiang Uygur Autonomous Region
7	Quanzhou Women and Children’s Hospital
8	Children’s Hospital of Zhejiang University
9	Xiamen Maternity and Child Health Care Hospital
10	Xiamen Children’s Hospital
11	Shenzhen Hospital of Hongkong University
12	Qingdao Women and Children’s Hospital
13	First Affiliated Hospital of Kunming Medical University
14	Guizhou Women and Children’s Hospital
15	Suzhou Municipal Hospital Affiliated to Nanjing Medical University
16	The Second XiangYa Hospital of Central South University
17	Qilu Children’s Hospital of Shandong University
18	Hebei Children’s Hospital
19	Hunan Children’s Hospital
20	The First Affiliated Hospital of Xinjiang Medical University
21	Xinhua Hospital Affiliated to Shanghai Jiaotong University School of Medicine
22	Obstetrics & Gynecology Hospital of Fudan University
23	Foshan Women and Children’s Hospital
24	Children’s Hospital of Shanghai
25	Shaanxi Provincial People’s Hospital
26	Wuxi Maternity and Child Healthcare Hospital
27	Shenzhen Children’s Hospital
28	Henan Children’s Hospital
29	The Affiliated Hospital of Qingdao University
30	Gansu Provincial Maternity and Child Care Hospital
31	The First Affiliated Hospital of Zhengzhou University
32	Children’s Hospital of Soochow University
33	Yuying Children’s Hospital Affiliated to Wenzhou Medical University
34	Jiangxi Provincial Children’s Hospital
35	Dalian Municipal Women and Children’s Medical Center
36	Children’s Hospital Affiliated to Capital Institute of Pediatrics
37	General Hospital of Ningxia Medical University
38	Anhui Provincial Hospital
39	Guangzhou Women and Children’s Medical Center
40	Beijing Children’s Hospital of Capital Medical University
41	The Third Affiliated Hospital of Zhengzhou University
42	Fuzhou Children’s Hospital of Fujian Province
43	Fujian Women and Children’s Medical Center
44	Children’s Hospital of Nanjing Medical University
45	The First Affiliated Hospital of Anhui Medical University

46	Hainan Women and Children’s Hospital
47	Children’s Hospital of Fudan University
48	Women and Children’s Hospital of Guangxi Zhuang Autonomous Region
49	Shanghai First Maternity and Infant Hospital
50	Northwest Women’s and Children’s Hospital
51	Tianjin Obstetrics & Gynecology Hospital
52	Lianyungang Maternal and Children Health Hospital
53	Shanghai General Hospital
54	Inner Mongolia Maternal and Child Health Care Hospital
55	Jiangsu Women and Children Health Hospital
56	Ningbo Women & Children Hospital
57	Maternal and Children Hospital of Shaoxing
58	Changzhou Maternal and Children Health Care Hospital
59	Anhui Children’s Hospital
60	Chongqing Health Care Center for Women and Children
61	The First People’s Hospital of Yunnan Province
62	The Affiliated Hospital of Southwest Medical University
63	Wuhan Children’s Hospital
64	The Second Hospital of Hebei Medical University
